# Let’s Play at Digging: How Vigorous Is This Energetic Task for a Young Forager?

**DOI:** 10.1007/s12110-022-09428-w

**Published:** 2022-06-06

**Authors:** Ana Mateos, Guillermo Zorrilla-Revilla, Jesús Rodríguez

**Affiliations:** National Research Center On Human Evolution (CENIEH), 09002 Burgos, Spain

**Keywords:** Digging, Energetics, Foraging, Childhood, Adolescence

## Abstract

Extractive foraging tasks, such as digging, are broadly practiced among hunter-gatherer populations in different ecological conditions. Despite tuber-gathering tasks being widely practiced by children and adolescents, little research has focused on the physical traits associated with digging ability. Here, we assess how age and energetic expenditure affect the performance of this extractive task. Using an experimental approach, the energetic cost of digging to extract simulated tubers is evaluated in a sample of 40 urban children and adolescents of both sexes to measure the intensity of the physical effort and the influence of several anatomical variables. Digging is a moderately vigorous activity for inexperienced girls and boys from 8 to 14 years old, and it requires significant physical effort depending on strength and body size. However, extracting subterranean resources is a task that may be performed effectively without previous training. Sex-specific and age-specific differences in the net energy expenditure of digging were detected, even though both sexes exhibited similar proficiency levels when performing the task. Our results highlight that both boys and girls spend considerable energy while digging, with differences largely driven by body size and age. Other factors beyond ability and experience, such as strength and body size, may influence the proficiency of juveniles in performing certain physically intensive foraging tasks, such as gathering tubers.

**Supplementary Information:**

The online version contains supplementary material available at 10.1007/s12110-022-09428-w.

Childhood and adolescence are complex stages of human life history (Leigh, 2001; Walker et al., 2006b) dedicated to brain development as well as somatic growth and maintenance to achieve adult body size (Bogin, 1997, 1998) and to support the onset of sexual maturation (Kotler & Haig, 2018). These stages have high energetic costs to allocate growth, immunological defense, and somatic maintenance (Kramer & Greaves, 2011; Torun, 2005; Urlacher et al., 2019). During childhood and adolescence, young people begin to learn, train, and acquire the subsistence and reproductive skills (mating, parenting, and providing care, among others) necessary to perform their adult roles (Kramer, 2021; MacDonald & Hewlett, 1999). In nonindustrial societies, small-bodied children expend little energy while training to acquire adult abilities, but simultaneously they begin to be productive—that is, they begin to participate in provisioning activities and domestic chores (Blurton Jones et al., 1989; Crittenden & Zes, 2015; Crittenden et al., 2013) despite being dependent on adults for subsistence. In Hazda, Mikea, and Aka societies, juveniles are expected to cooperate in the food economy, to be fairly self-sufficient, and they are expected to learn and practice skills, decision making, and social norms as they mature (Froehle et al., 2019; Lew-Levy et al., 2017; Tucker & Young, 2005). Moreover, adolescents acquire most of their body strength, body size, and psychomotor skills (Cromer et al., 2015) to practice adult activities, while still being far from the peak of their net productive energy (Gurven & Walker, 2006; Walker et al., 2002). As such, an extended period of juvenile dependency also has fitness costs.

In fact, many scholars state that child and adolescent hunter-gatherers are mainly dependent on adults for subsistence because they need a long apprenticeship and substantial training in foraging tasks and reproductive skills; this perspective assumes that juveniles contribute little to their own maintenance (Bogin, 1998; Gurven & Kaplan, 2006; Gurven & Walker, 2006; Kaplan, 1996; Kaplan et al., 2001). However, other authors have theorized that children and adolescents begin to be productive for themselves and for the group while still being dependent on adults for their own survival (Bird & Bliege Bird, 2005; Blurton Jones & Marlowe, 2002; Blurton Jones et al., 1989; Codding et al., 2011; Crittenden & Zes, 2015; Crittenden et al., 2013; Draper, 1976; Draper & Cashdan, 1988; Kramer, 2002, 2005; Robinson et al., 2008). This debate over juveniles’ broad dependency on adults and the contribution of young foragers to their own subsistence is ongoing. Many of the hypotheses in the literature are based on a number of theories about life history, which emphasize the impact of experience and learning as powerful selective forces to explain the slow growth and the delayed juvenility of humans (Bock, 2005; but see Kramer, 2021). Kaplan et al. (2000) highlight the lengthy learning and training time needed to acquire adult abilities based on the idea that human foraging is skill-intensive. Time spent being juveniles increases their subsequent energy production as adults and corresponds to an increase in embodied capital related to learning and growth (Charnov, 1993, 2004; Gurven & Walker, 2006; but see Blurton Jones & Marlowe, 2002). As a result, children and adolescents are primarily reliant on the foraging success of adults (Kaplan et al., 2001). Another hypothesis, the punctuated developmental model (Bock, 2002), integrates the influence of experience factors of learning, task-specific abilities, or cognitive functions with other traits derived from growth (such as body size, strength, and coordination). The punctuated developmental model is an interesting theoretical refinement because it combines the physical attributes and the specific behaviors and abilities of juvenile hunter-gatherers to gain experience performing foraging tasks.

Undoubtedly, juvenile hunter-gatherers must learn numerous foraging skills, and it takes them many years of experience to reach their peak productivity (Koster et al., 2020). As may be expected, this continued practice improves the performance of a task. Koster et al. (2020) pointed out that skillfulness peaks after physical and reproductive maturation, and thus the proficiency in some foraging activities accelerates during childhood and adolescence. Some of the aforementioned studies assumed that children require provisioning from adults until their adolescence and identified the time needed for learning as the main growth-based constraint (Kaplan et al., 2000; Schuppli et al., 2012). Some recent works reject this claim by highlighting that juveniles are not only dependent consumers but also producers, even from very young ages (Bird & Bliege Bird, 2005; Tucker & Young, 2005). Other studies (Bird & Bliege Bird, 2002; Blurton Jones & Marlowe, 2002) found that differences in physical traits such as body size and strength (Apicella, 2014; Bird & Bliege Bird, 2005; Kelly, 2013; Kramer & Greaves, 2011) have stronger effects on specific activities than age-related experience (see Bock, 2002; Ohtsuka, 1989). In hunter-gatherer populations, children and adolescents clearly participate in the economic activities of the group and in their self-provisioning (Kramer & Ellison, 2010, and references therein). Additionally, sex-specific tasks are evident in the daily routine of juveniles in foraging societies that inhabit different environments, wherein boys and girls simulate foraging activities and productive chores while playing (Apicella et al., 2017; Crittenden, 2016; Crittenden et al., 2013; Froehle et al., 2019; Gallois et al., 2015; Keith, 2006; Lew-Levy et al., 2017; Zorrilla-Revilla et al., 2021). Indeed, it has been widely reported that juveniles cooperate in foraging from an early age (Blurton Jones et al., 1994; Crittenden et al., 2013; Hawkes et al., 1995; Lew-Levy et al., 2017, 2018).

The strong sexual division of labor among the Hadza arises in middle childhood, although their young children forage and play in mixed-age and mixed-sex groups (Hawkes et al., 1995), and the sex differences become more pronounced during adolescence (Crittenden et al., 2021; Froehle et al., 2019). Pumé girls occasionally accompany women during their foraging trips, but they often perform tasks in the camp such as caring for siblings, collecting water, and other domestic chores (Kramer & Greaves, 2011). It might be argued that since the sexual division of tasks starts in middle childhood and the beginning of puberty (Crittenden et al., 2021; Zorrilla-Revilla et al., 2021), ontogenetic differences in the size and strength of juveniles might be influencing these differences in their daily routine. Additionally, child and adolescent foragers may improve their parents’ fitness as well as their own individual fitness (Lee & Kramer, 2002; Lew-Levy et al., 2017, 2020; Nag et al., 1978) by producing a surplus of energetic return that can be shared with siblings and parents (Bird & Bliege Bird, 2002, 2005; Kramer, 2002, 2005, 2018; Kramer & Ellison, 2010; Lee & Kramer, 2002; Reiches et al., 2009). !Kung and Hadza children can collect between 50 and 100% of their daily energy requirements, depending on sex, age, and the type of resource targeted (Blurton Jones et al., 1994, 1997; Crittenden et al., 2013; Draper & Cashdan, 1988; Froehle et al., 2019; Hawkes et al., 1995; Robinson et al., 2008). Therefore, the improvements in, and proficiency of, foraging skills with age are not likely to be exclusively a result of training. Progress in the acquisition of skills may also be due to changes in body size and other physical traits of the young foragers that can increase the strength directed toward the subsistence task and influence the energy expended to perform the task (Bock, 2005). Moreover, depending on the physical demands, the complexity of the task, and the technology required to do it competently, young foragers may be able to extract and obtain resources from the environment in an efficient manner.

In any case, although foraging must be learned, mastering some of the related tasks is often easy and quick. For example, certain resources can be gathered with great ease. Thus, some gathering tasks may be performed without problems, provided that adequate resources are available nearby, items are easy to locate, and there is no competition for the resource. In this case, juveniles may spend several hours every day collecting food (Blurton Jones & Marlowe, 2002; Crittenden et al., 2013; Froehle et al., 2019; Tucker & Young, 2005) in addition to playing, taking care of their younger siblings, collecting firewood and water, cooking, washing, and other in-camp tasks in mixed-age and mixed-sex groups (Kramer & Veile, 2018). Time allocation of juveniles to foraging tasks differs according to environmental constraints and the social structure of the hunter-gatherer group. Hadza children devote considerable amounts of time to leisure, resting, and playing (Froehle et al., 2019). However, as they mature they spend less time playing and more time in work-related activities around the camp, such as collecting water, maintaining living spaces, and performing more difficult food processing tasks. Moreover, their energetic contributions to subsistence tasks begin to show sex differences at an early age (Apicella et al., 2017; Froehle et al., 2019). This pattern is similar to other small-scale societies, such as Mikea (Tucker & Young, 2005), Aka (Lew-Levy & Boyette, 2018), and other foragers (Lew-Levy et al., 2017). Mikea juveniles allocate a remarkable amount of time every day to tuber foraging tasks. Adolescents of both sexes spend around 10% of their time performing this task; females devote 12.4% and males, 8.1%. In contrast, children of both sexes allocate around 66% of their daily time to leisure activities such as playing, and around 5% of their time foraging for tubers; females devote 4.9% and males, 5.6% (see Tucker & Young, 2005: figs. 7.2 and 7.3). Pumé young girls spend on average about 5% of the daylight hours in domestic activities, and girls aged 7 to 10 years allocate 2% of their time on average in foraging activities and 12% of daylight hours in domestic tasks (Kramer & Greaves, 2011).

Gathering is one of the most common foraging activities among juvenile hunter-gatherers (Blurton Jones et al., 1989, 1997; Crittenden et al., 2013; Froehle et al., 2019; Hawkes et al., 1995), and it is performed by both girls and boys of many ages (i.e., up to middle childhood, when male and female foragers begin targeting sex-specific productive activities) (Crittenden et al., 2013). Gathering is a very profitable activity (Tucker & Young, 2005), and it may be performed alongside adults (Hawkes et al., 1995) or in sex-based peer groups (Crittenden, 2016; Crittenden et al., 2013). However, successful gathering of food resources does not necessarily require cumulative experience. Indeed, Mardu and Meriam children are efficient collectors of small prey and marine resources (Bird & Bliege Bird, 2002, 2005; Bliege Bird & Bird, 2002). According to Lew-Levy et al. (2021), the difficulty of a task is not related to the age of skill acquisition; Bird and Bliege Bird (2002, 2005) highlighted that body size rather than skill seems to limit the energetic return of foraging.

With these premises in mind, we consider that other factors beyond ability and experience can influence the proficiency of juveniles in performing certain foraging tasks. Therefore, extractive foraging such as digging may be an ideal task to analyze since it is broadly practiced among hunter-gatherer populations across different ecological conditions (Blurton Jones et al., 1997; Blurton Jones & Marlowe, 2002; Marlowe & Berbesque, 2009; Tucker & Young, 2005 and references therein; Vincent, 1985). Tuber-gathering and extractive foraging are assumed to be labor intensive and physically demanding activities (Apicella et al., 2017; Blurton Jones & Marlowe, 2002; Crittenden et al., 2013). In fact, tuber-gathering is a task that requires low levels of skill and considerable strength (see Kramer, 2021, Fig. 1). The strength-based versus skill-based difficulty of performing a subsistence task should be considered alongside the ontogenetic limitations by age and sex in the skillfulness of child and adolescent foragers. Based on the aforementioned premises, although digging does not seem to be a difficult skill to acquire or practice (Blurton Jones & Marlowe, 2002), it is presumably energetically expensive, and the energy expenditure is likely related to the strength and body size of the individual, which are both closely related to age. Nevertheless, little research has examined the physical traits associated with digging ability (e.g., the strength of the upper body), which are likely relevant for performing this task (Apicella et al., 2017; Kramer & Greaves, 2011). The aim here is to evaluate the energetic cost of digging for extracting resources using an experimental approach with novice diggers. The energy expenditure of a simulated extractive foraging activity (digging) was measured in a sample of 40 urban children and adolescents of both sexes to assess the intensity of the physical effort and to evaluate the influence of anatomical variables related to their growth and maturation. Undoubtedly, a mismatch between foraging societies and contemporary industrialized societies exists (Kramer, 2021, and references therein) when differences in lifestyle, nutritional adequacy, access to health services and food supplies, family and/or group structure, schooling, and even growth and developmental rates are considered. Children in industrialized populations grow faster and have more stable patterns of development. Furthermore, a secular trend of increasing height and weight related to urbanization is known (Bogin, 1999; Eveleth & Tanner, 1990), and better socioenvironmental conditions have been associated with faster child and juvenile growth rates and with an earlier adolescent growth spurt (McCullough & McCullough, 1984; Stinson, 2000; Walker et al., 2006a). However, we are confident in the suitability of our experimental approach, with urban children and adolescents reenacting a foraging task.

## Methods and Analyses

### Participants

Volunteers were recruited by distributing flyers in local schools and through advertisements in local media and CENIEH (Centro Nacional de Investigación sobre la Evolución Humana/National Research Center on Human Evolution) social networks. Mail distribution lists from the LabBioEM (BioEnergy Laboratory) facility were also used. The sample was composed of 40 individuals (23 males and 17 females between 8 and 14 years of age) who were in middle childhood and early adolescence. All children and adolescents recruited were white residents of Burgos, a medium-sized city in the north of Spain. They are healthy individuals (i.e., not overweight or obese) from middle socioeconomic status families. Parental socioeconomic status was not established on the basis of a standardized survey, but based on the fact that all participants live in urban middle-class residential areas. The age interval was chosen on the basis of ethnographic studies (Hewlett, 2016), which consider the end of middle childhood as the period when individuals adopt productive activities in most subsistence-based societies. Although our experimental study was performed with urban children, our methods are valid and suitable to detect the energetic differences between boys and girls when performing a simulated tuber-gathering task. Although our results cannot be directly translated to the juveniles of a foraging society, they do reflect the effect of ontogenetic and physical limitations on the daily activities performed at those young ages.

### Experimental Design

The experimental study was approved by the Hospital Universitario de Burgos Ethical Committee (Burgos, Spain) (BioE5-CEIC 1586), and the ethical guidelines were followed by the research team, which was led by A. Mateos. Data collection was carried out between 2016 and 2017. Prior to data acquisition, written informed consent was obtained from the participants and their legal guardians. The sample was exhaustively controlled using exclusion criteria that aimed to eliminate certain influencing factors of metabolic cost, such as medication, food ingestion, or metabolic and cardio-respiratory pathologies. Prior to performing the trials, all volunteers were required to fast overnight to avoid the thermic effect of food on Energy Expenditure (EE). The experimental design was carried out in two different sessions, one indoors and another outdoors.

The first session was conducted indoors in the BioEnergy Laboratory at CENIEH (Burgos, Spain). Here, standardized protocols were applied to all individuals. Participants were anthropometrically characterized based on the normalized standards of Lapunzina and Aiello (2002). Body mass (BM) was measured with a digital scale to the nearest 0.1 kg, height (H) was measured to the nearest to 0.1 cm with a Harpenden stadiometer (Holtain Ltd.), and body segments were measured by means of a Harpenden anthropometer and an anthropometric tape. Body segments related to the upper limb were measured in the dominant arm of each individual, and upper limb length (UpLL) was taken from the most superior lateral point of the acromion process (acromio-clavicular joint) to the end of the third finger. To this end, the arm was positioned at the highest extension in the anatomical position and relaxed at the side of the subject. We also computed the brachial index as [(RL*100) / HL], where RL is radius length and HL is humerus length (Mateos et al., 2019).

Body composition protocol was performed using a Bioelectrical impedance vector analysis BIA 101 AKERN® and BodyGram Pro^©^ software (v2010), in accordance with the standardized protocol established in the Consensus Conference of the National Institutes of Health (1996). The body composition variables obtained for each individual were fat mass (FM), fat-free mass (FFM), and muscle mass (MM) (in kilograms). Metabolic rates were measured by breath-by-breath ventilatory indirect calorimetry protocols, which were monitored through oxygen consumption and carbon dioxide production using a Master Screen-CPX JAEGER® device, and then analyzed by the LabManager IntelliSupport 5.72 application. The milliliters (ml) of O_2_ and CO_2_ and the equivalent metabolic rate in kilocalories (kcal) (Weir, 1949) were then recorded. The resting metabolic rate (RMR) was quantified over a 30-min period, with each participant laying on a stretcher while wearing a breathing mask and a heart rate monitor.

**Fig. 1:**
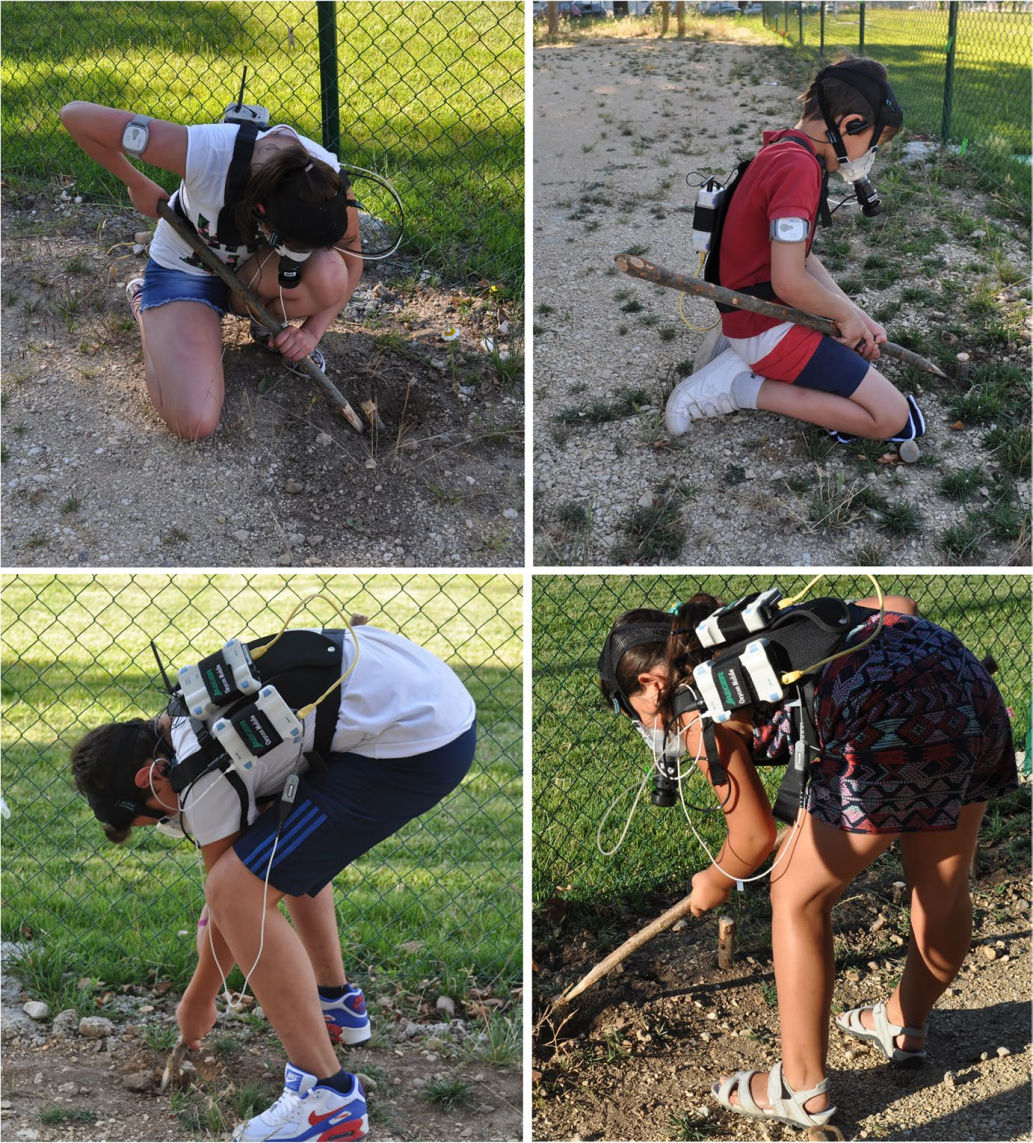
Volunteers simulating digging tasks with the help of wooden sticks. Energy expenditure was monitored using an Oxycon Mobile JAEGER.® portable device

The second session was performed outdoors, where a digging activity was developed to emulate the performance of a tuber-gathering task. For the outdoor trials, energy expenditure was monitored using an Oxycon Mobile JAEGER® portable device. The calorimetry device has been lab-validated as criterion standard system (Akkermans et al., 2012; Sjöberg et al., 2021) and has been used in previous field experiments in reenacted activities (Mateos et al., 2019; Prado-Nóvoa et al., 2017; Vidal-Cordasco et al., 2017; Zorrilla-Revilla et al., 2021). All of the ventilatory tests, both indoors and outdoors, were performed under standard environmental conditions of temperature, barometric pressure, and relative humidity, calibrated automatically using ambient STPD conditions (standard temperature, pressure, and dryness). In a second step, for an exact determination of lung volume (V), the measuring system of the JAEGER® portable device (Triple V) was calibrated, and finally, the gas analyzers (O_2_/CO_2_) integrated in the device were calibrated by means of gas cylinders containing 5% CO_2_ and 16% O_2_. The digging trial consisted of finding and digging up wooden stakes simulating shallow tubers (which had been buried by the research team), with the help of a wooden stick (Fig. 1). Twenty-five simulated tubers were buried to a depth of 10–20 cm. The wooden digging sticks ranged in length from 74.4 to 101.5 cm, with an average of 84.40 cm (as reported among Australian Aborigines and archaeologically documented at La Draga; López-Bultó et al., 2020, and references therein). The digging took place for a 15-min period, during which participants did not rest at any time (see sample output from the experimental trial in Figure S1, Electronic Supplementary Material). The LabManager IntelliSupport 5.72 application of Oxycon Mobile JAEGER® computes and reassesses the variation in the metabolic rate during the test to better evaluate the energy expenditure. Participants did not receive any specific instructions on how to perform the simulated tuber-gathering task. The research team just asked each participant to choose one of the three wooden sticks and to use it to dig out the buried stakes. Participants were not given clues about the exact position of the buried stakes. In a few cases, participants who were siblings had the opportunity to observe their brother or sister complete the task before performing it by themselves.

The energy expenditure data were standardized to kilocalories per hour (kcal/h) and per day (kcal/day). Energetics of digging (E_dig_; variable equivalent to the energy cost of foraging, E_f_, in Kraft et al., 2021) was expressed as the gross cost of digging (DIG_Gross_) and the net cost of digging (DIG_Net_). The net cost of an activity is obtained by subtracting the resting metabolic rate from the measured gross cost of the activity. Furthermore, all energetic data were translated into MET (Metabolic Equivalent Task of Intensity) using the equation MET = EE / (Body Mass * Time), where energy expenditure is measured in kcal, body mass in kg, and time in hours. In accordance with the Compendium of Physical Activities (Ainsworth et al., 2000) and the World Health Organization report (WHO, 2010), all test results were expressed in MET. All of the data were then incorporated into the EVOBREATH Database (Mateos et al., 2016). Data that support the findings of this study are available in an online dataset of Mendeley Data (Mateos & Rodríguez, 2021).

### Statistical Analyses

Statistical tests were run using Dell® Statistica 13 Academic. For statistical purposes, all individuals were classified into one of three age groups: group 1 (from 8 to 10 years old), group 2 (from 11 to 12 years old), and group 3 (from 13 to 14 years old). These age groups were established before starting the recruitment of volunteers since they represent three different stages of development: children, preadolescents, and early adolescents. We did not use radiologic analyses nor any other invasive technique to determine the developmental stages. ANOVA tests were used to test for differences in the main variables (stature, body mass, fat mass, fat-free mass, muscular mass and metabolic rates, RMR, DIG_Gross_, and DIG_Net_) by age group and sex. The efficiency of digging was examined by considering the net energy expenditure (cost) of the task and the number of stakes (return) extracted by each participant. The efficiency index (EI) was computed by dividing DIG_Net_ by the number of stakes dug out by each individual. Note that the return concept used here is based on the number of stakes achieved during the extractive task, and it is not equivalent to the caloric return of the items (E_a_, energy acquired, in Kraft et al., 2021). An ANOVA test was used to examine the effects of age group and sex factors on EI.

## Results

The main anthropometric measurements and metabolic rates of the individuals in the sample are listed in Table 1. ANOVA tests show significant differences in all the anthropologic variables by age, and a significant interaction of age and sex in all cases (Table 2). Indeed, age covaries with many body variables linked with growth and maturation, and such differences among age groups are noticeable. Even though girls generally exhibited lower values than boys for many of the variables, in age group 2 (11–12 years old) girls were taller and had a higher body mass than their male counterparts. As expected, girls had a higher proportion of fat mass from 11 to 14 years old (Vink et al., 2010; Wells, 2007). Interestingly, the value of fat-free mass and muscular mass in girls of age group 2 was higher than for boys, which may be related to the higher body-size (in stature and body mass) seen in girls this sample. Moreover, their upper limb length was longer than boys’ in this age cohort, but shorter in the other age groups (1 and 3) (Table 1). In the sample, the brachial index was higher in boys than in girls in all groups. As expected, the anatomical variables of the upper limb, such as UpLL and the brachial index, are significantly correlated with the net cost of digging (DIG_Net_) (UpLL, *p* = 0.58; BI, *p* = 0.37; *p* < 0.05).

**Table 1:** Mean and standard deviation (in parentheses) of body parameters and metabolic rates of the volunteers participating in the tests by age group and sex. GrossCOT includes the resting metabolic rate and the activity cost. UpLL is Upper limb length, Brachial Index is computed as [(Radius Length*100) / Humerus Length], RMR is the Resting Metabolic Rate, DIG_Gross_ is the gross cost of digging, and DIG_Net_ is the net cost of digging

	Age group 1 (8–10 years)			Age group 2 (11–12 years)			Age group 3 (13–14 years)		
	Male	Female	Total	Male	Female	Total	Male	Female	Total
*N*	11	7	18	8	5	13	4	5	9
Stature (cm)	138 (7.49)	133.45 (9.20)	136.23 (8.25)	151.53 (7.21)	160 (4.81)	154.79 (7.51)	167.7 (7.80)	158.76 (7.00)	162.73 (8.34)
Body Mass (kg)	31.03 (5.16)	28.41 (6.67)	30.01 (5.75)	42.83 (8.88)	55.84 (7.15)	47.83 (10.31)	57.1 (7.76)	46.62 (7.77)	51.27 (9.12)
UpLL (cm)	63.02 (4.35)	59.51 (3.97)	61.66 (4.45)	67.71 (4.50)	74.48 (2.36)	70.31 (5.04)	77.4 (5.06)	72.24 (4.34)	74.53 (5.14)
Brachial Index (cm)	66.20 (4.32)	61.78 (3.70)	64.48 (4.56)	65.38 (3.91)	64.15 (1.65)	64.91 (3.20)	68.61 (6.03)	61.03 (4.61)	64.40 (6.34)
Fat Mass (kg)	6.55 (3.19)	6.75 (4.13)	6.63 (3.47)	7.51 (4.09)	15.38 (4.70)	10.53 (5.74)	8.47 (2.96)	9.58 (3.35)	9.08 (3.04)
Fat-Free Mass (kg)	24.52 (4.68)	21.61 (3.97)	23.39 (4.54)	35.32 (5.99)	40.46 (2.69)	37.3 (5.49)	48.62 (8.64)	37.04 (5.43)	42.18 (8.94)
Muscular Mass (kg)	12.96 (2.13)	11.6 (2.15)	12.43 (2.19)	19.23 (3.13)	23.1 (5.98)	20.72 (4.63)	28.35 (6.19)	19.8 (2.04)	23.6 (6.06)
RMR (kcal/d)	1,607.18 (203.19)	1,467.85 (171.71)	1,553 (198.94)	1,294.87 (256.69)	1,610.2 (285.59)	1,416.15 (301.85)	1,649.25 (399.54)	1,528.2 (413.09)	1,582 (386.33)
DIG_Gross_ (kcal/h)	180.59 (49.03)	182.02 (32.81)	181.14 (42.36)	251.93 (63.11)	228.52 (59.35)	242.93 (60.32)	374.54 (180.77)	204.14 (65.99)	279.87 (149.99)
DIG_Net_ (kcal/h)	113.62 (47.05)	120.86 (29.93)	116.43 (40.39)	197.98 (55.76)	161.43 (65.96)	183.92 (60.05)	305.82 (168.22)	140.46 (57.26)	213.95 (140.88)

**Table 2:** ANOVA tests for the main body variables by sex and age groups. FM is Fat Mass, FFM is Fat-Free Mass, MM is Muscular Mass. The asterisk indicates significant differences

		Intercept	Sex	Age Group	Sex*Age Group	Error
Stature	SS	819,920.1	25.0	5369.8	467.9	1906.3
	df	1	1	2	2	34
	F	14,623.71	0.45	47.89	4.17	
	p	< 0.0001*	0.5089	< 0.0001*	< 0.05*	
Body Mass	SS	67,968.6	0.01	4078.3	790.5	1714.3
	df	1	1	2	2	34
	F	1347.99	0.00	40.44	7.83	
	p	< 0.0001*	0.9889	< 0.0001*	< 0.01*	
FM	SS	2918.5	83.4	165.2	114.2	482.4
	df	1	1	2	2	34
	F	205.71	5.88	5.82	4.02	
	p	< 0.0001*	< 0.05*	< 0.0001*	< 0.05*	
FFM	SS	42,719.7	86.9	2827.9	363.2	937.1
	df	1	1	2	2	34
	F	1549.83	3.15	51.30	6.59	
	p	< 0.0001*	0.0847	< 0.0001*	< 0.01*	
MM	SS	13,121.7	36.30	1010.51	198.80	417.4
	df	1	1	2	2	34
	F	1068.90	2.95	41.16	8.09	
	p	< 0.0001*	0.0946	< 0.0001*	< 0.01*	

Concerning the metabolic rates of the individuals in the current sample (Table 1), the overall RMR was higher in males than females except for age group 2, where girls had higher values of RMR than boys, likely due to the larger body size and higher proportion of fat-free mass in this age cohort. The gross and net cost of digging was higher in males than females, except in age group 1, where girls had similar DIG_Gross_ values. The results show that in individuals aged 10 years or more, DIG_Net_ was, generally speaking, greater in boys than in girls (ESM, Figure S1). Concerning the physical effort and the energy expenditure of digging, this is a moderate activity for the average individual in the sample (mean = 5.70 MET; *SD* = 1.80), according to Ainsworth et al. (2000). However, considering the differences in sex, digging was, on average, classified as a vigorous activity (> 6 METs) for boys (mean = 6.06 MET; *SD* = 1.90), but a moderate activity for girls (mean = 5.22 MET; *SD* = 1.58).

**Fig. 2:**
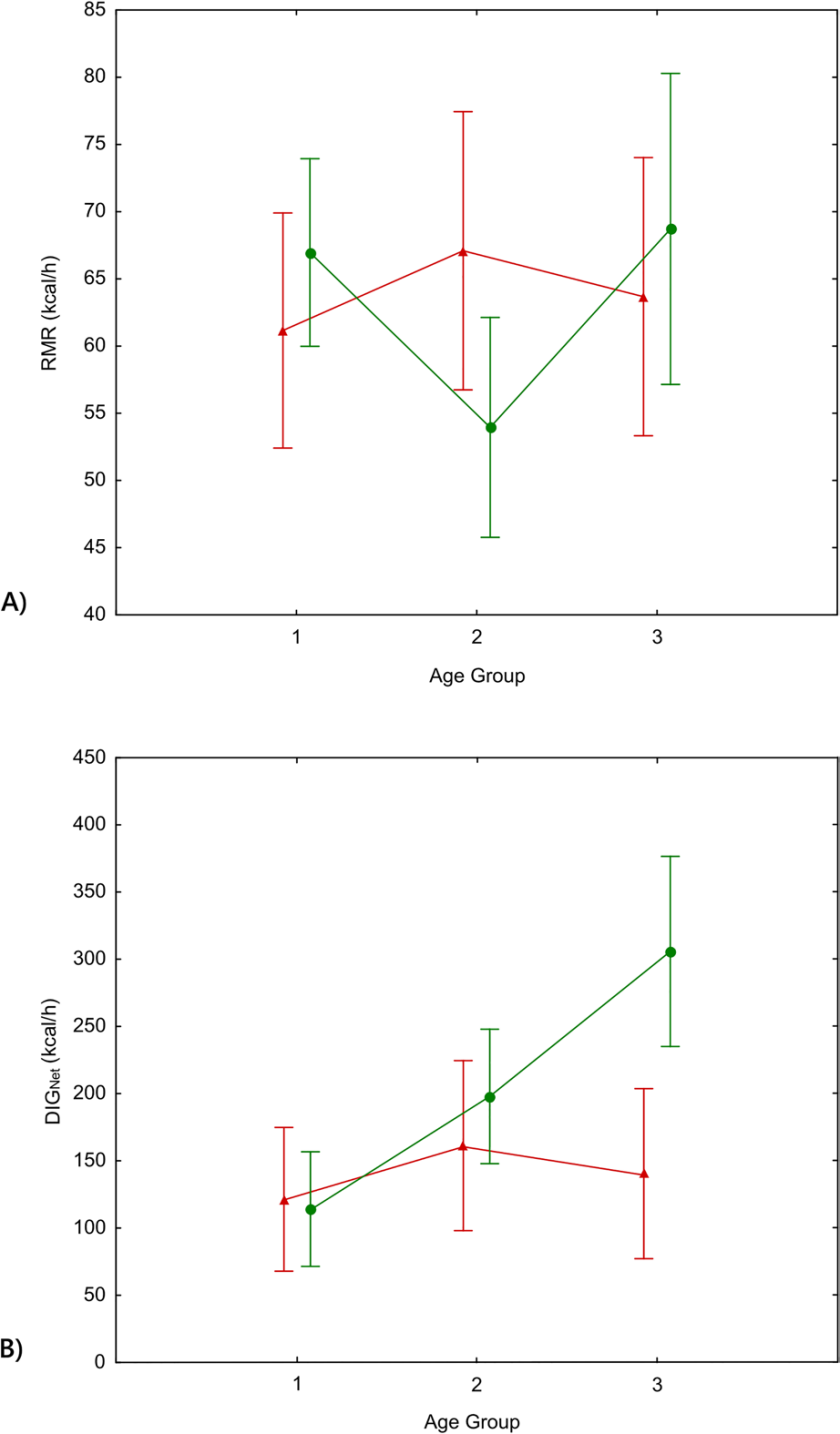
Energy expenditure by sex and age group. **A** Resting metabolic rate (RMR) and **B** net cost of digging (DIG_Net_). The vertical bars represent the 95% confidence interval. Age groups: group 1 (from 8 to 10 years old), group 2 (from 11 to 12 years old), and group 3 (from 13 to 14 years old)

The ANOVA test showed no statistically significant differences in RMR based on age cohort or sex. This is an unexpected result because it is well established that RMR is correlated to body size (Kleiber, 1947; White & Seymour, 2003), and body size increases with age and covaries with sex (Table 2). In contrast, the cost of digging did reveal differences based on age and sex (Table 3, ESM Figure S1). A post-hoc test (Tukey’s procedure) showed that DIG_Net_ differed significantly between boys and girls in the oldest cohort, and between the youngest and oldest age cohorts of boys (Fig. 2).

**Table 3:** Analysis of variance (ANOVA) in the metabolic rates RMR and DIG_Net_, categorizing by sex, age group and Sex*Age group. The asterisk indicates significant differences

	RMR				DIG_Net_			
	SS	df	F	p	SS	df	F	p
Intercept	144,326.8	1	1113.33	< 0.0001*	1,072,604	1	221.41	< 0.0001*
Sex	5.2	1	0.04	0.8425	37,567	1	7.75	0.0087*
Age Group	179.0	2	0.69	0.5083	71,367	2	7.37	0.0022*
Sex*Age Group	729.8	2	2.81	0.0739	44,049	2	4.55	0.0178*
Error	4407.6	34			164,709	34

The number of stakes retrieved varied widely between individuals. Two participants failed to recover any, but the average number of stakes dug out was 5.8, and one individual managed to extract 21 items. Though boys had higher net energetic cost of digging than girls, and also a higher mean EI, which indicates lower efficiency (EI = 9.81 kcal/stake for boys and EI = 7.73 kcal/stake for girls), the differences in efficiency by sex were not statistically significant (Table 4). Moreover, the differences between age groups and the interaction of the two factors are also nonsignificant (Table 4).

**Table 4:** Analysis of variance (ANOVA) for the efficiency index (EI) by sex, age group, and Sex*Age group. The asterisk indicates significant differences

	EI			
	SS	*df*	F	*p*
Intercept	2521.9	1	75.06	< 0.0001*
Sex	37.2	1	1.11	0.3005
Age Group	7.5	2	0.11	0.8943
Sex*Age Group	31.6	2	0.47	0.6290
Error	1075.2	32		

## Discussion

Our results show that digging is a moderately vigorous activity for girls and boys aged 8–14 years and, thus, requires significant physical effort. Although both girls and boys exhibited considerable energy expenditure during the task, sex-specific and age-specific differences in the net cost of digging were detected. The anthropometrics of the individuals in the sample differ by sex and age, but some distinct patterns are revealed when our sample is compared with data from forager children and adolescents (see Ford & Crittenden, 2017; Pollom et al., 2021 for Hadza foragers). Actually, !Kung children and adolescents (aged 7–14 years) are shorter and have less body mass than the individuals in our sample. As an example, the average stature of male !Kung juveniles aged 7 to 14 years is 120.5 cm (*SD* 10.28) and the body mass is 20.5 kg (*SD* 3.67) (Howell, 1979, 2010). In contrast, the average stature of the boys in our sample is 147.87 cm (*SD* 13.25) and the body mass is 39.67 kg (*SD* 11.90). In !Kung female juveniles in the same age cohort, the average stature is124.4 cm (9.34) and the body mass is 22.5 kg (4.43) (Howell, 1979, 2010), while the average height of the girls in our sample is 148.71 cm (*SD* 14.93) and the average weight is 41.84 kg (*SD* 13.85). In summary, the urban children and adolescents in our sample are markedly larger (in stature and body mass) than the !Kung juveniles in the same age cohort. Concerning the proportions of the arms, our participants’ brachial index is higher in boys from age group 1 to age group 3 than in girls. This index reflects the proportions of the forelimb, and it has been shown to influence the energetic cost of certain tasks involving the upper limb, such as stone knapping (Mateos et al., 2019). Thus, the brachial index might also be expected to affect the energetic cost of digging. Indeed, the sexual dimorphism affecting the limbs is a well-known feature in humans (Cowgill et al., 2012; Temple et al., 2011). The human sexual dimorphism affecting the strength of the upper body is maintained in adulthood and is also present in younger children, despite not being well understood (Apicella, 2014).

As previously mentioned, participants received no instructions on how to use the wooden stick to extract the simulated tubers. However, most of them managed to obtain several stakes despite this being an entirely new activity for them. In other words, although the skill was not trained in any way, most participants performed the task with remarkable success. Most kids did not see other participants digging, and all of them were untrained subjects. Furthermore, the postures and technical gestures of the participants (Fig. 1), which ranged from standing or bending to kneeling, are not the usual postures adopted by hunter-gatherers while digging. Indeed, the ethnographic record shows that adults and juvenile foragers sit down or kneel when digging (Kramer, 2021; Vincent, 1985; but see postures of chimpanzees in an experimental study by Motes-Rodrigo et al., 2019). The ergonomics of postures and the efficiency of the arm movements may certainly influence the efficacy of the technical gestures of digging and the physical effort required. However, analyzing those biomechanic and kinematic factors is beyond the scope of this experiment. Despite the different energy expenditure of girls and boys, they exhibited similar proficiency in digging. Therefore, it is unclear whether this ability is a result of individual motivation to participate in a scientific experiment or a by-product of their individual strength or skill. These results confirm earlier predictions on the foraging proficiency of juveniles in some small-scale societies (Hadza, Mikea, Martu, among others) (Bird & Bliege Bird, 2005; Blurton Jones & Marlowe, 2002; Crittenden et al., 2013; O’Connell & Hawkes, 1981; Tucker & Young, 2005). That is, body size differences have stronger effects than age and experience alone even though body size is notably related to age and sex, especially during middle childhood and early adolescence.

We are aware of some limitations of our study. First, the volunteers came from an industrialized and urban society and, therefore, were not familiar with the subsistence activities performed by hunter-gatherer groups. Although this is clearly an initial limitation, the general success of the performers in obtaining the simulated tubers suggests that the digging activity does not require specific knowledge. It should be acknowledged, however, that the skill to obtain tubers from a natural environment likely depends on knowledge about the distribution and location of resources (Blurton Jones et al., 1997; Crittenden et al., 2013; Vincent, 1985) rather than digging ability, as discussed below. Second, the digging trial was performed outdoors, but not in a natural environment; this could have influenced the behavior of the children and adolescents (Disma et al., 2011). Another limitation may be that our trial was not a reenactment of an excursion to forage and extract underground resources. Even though extractive foraging involves locating, digging, and carrying the obtained resources to the camp, participants only performed the task of digging, without any locomotion activities (Hagino & Yamauchi, 2014; Zorrilla-Revilla et al., 2021). Additionally, we could not measure the real energetic cost of such foraging trips, as this was beyond the scope of this study. However, this study is valid in terms of detecting the energetic differences between different sexes and age groups when performing the same activities, as in the case of other similar studies on adults and juveniles (Prado-Nóvoa et al., 2020; Zorrilla-Revilla et al., 2021).

Other drawbacks emerge when an experimental approach is carried out with urban kids from industrialized societies simulating foraging activities, as pointed out above. This mode of comparison identifies the potential mismatch between a hunter-gatherer lifestyle and a postindustrial one, as suggested by Kramer (2021). In fact, hunter-gatherer groups are not an analogy for the past; likewise, the urban populations are not an analogy for forager and traditional populations. However, the differences in the rate of development between urban and forager juveniles are partially accounted for in our approach because we considered three groups (children, preadolescents, and young adolescents) which may be correlated with similar developmental stages in the juveniles of foraging societies, even if they correspond to other chronological ages. Thus, we consider our approach to be sufficiently reliable to draw inferences and predictions about the constraints of some specific tasks requiring low-skill, high-strength, and high energetic requirements.

As highlighted by previous studies (Blurton Jones & Marlowe, 2002; Crittenden et al., 2013; Marlowe & Berbesque, 2009; O’Connell & Hawkes, 1981; Tucker & Young, 2005; Vincent, 1985), digging is relatively easy; the difficulty lies in locating the site where it should be performed. Often, young hunter-gatherer foragers go to these places accompanied by their parents and siblings (Kramer, 2011; Vincent, 1985). Therefore, juveniles can be both providers and dependents before they become net producers and proficient foragers in adulthood. In Hadza groups, they are fully proficient in terms of locating and extracting different species of tubers by the time they are 18–19 years of age (Crittenden et al., 2013). Furthermore, utilizing a very simple technology such as a wooden stick, young foragers are able to extract large amounts of an available and edible food source (including tubers, corms, bulbs, and rhizomes) using moderate energy output, while facing low risk and little pressure from competitors. These factors have made digging for vegetable resources an important foraging activity for humans and other primates for a long time (Hernández-Aguilar et al., 2007; Laden & Wrangham, 2005; Motes-Rodrigo et al., 2019; Truppa et al., 2019). This does not mean that a cumulative knowledge of certain skills is not necessary for the acquisition of this kind of resource, but proficiency in such an easy task may be acquired at an early age. Furthermore, Hadza children dig and scrape in the ground anytime and anywhere when they are playing, and they are able to obtain about 307 g/hr (around 163 kcal) by digging roots (Crittenden, 2016; Vincent, 1985). The return rates obtained ranged from 0.5 kg/hr (at 10 years old) to 0.8 kg/hr (at 12 years old) and 1.5 kg/hr (at 15 years old) to around 2 kg/hr (at 17 years old) (Blurton Jones & Marlowe, 2002, and references therein). Thus, digging may provide a relevant return even if the task is not performed at a high efficiency. Indeed, digging is not considered hard work by Hadza people, although strength and physical effort is required to perform this task (Blurton Jones et al., 1997; O’Connell & Hawkes, 1981; Vincent, 1985). Mikea children forage tubers with average net acquisition rates of 536 kcal/hr for girls and 504 kcal/hr for boys (Tucker & Young, 2005), whereas the average return rate of Hadza youths is 85 kcal/hr. These values may be compared with the estimated energy expenditure of digging in the experimental sample, which is roughly DIG_Net_ = 110–300 kcal/hr and DIG_Gross_ = 180–375 kcal/hr, on average. Conversely, Kaplan and collaborators (2000) state that Hiwi females achieve their complete root digging proficiency between the ages of 35 and 45, whereas Ache females reach peak proficiency of palm extraction in their early twenties (Tucker & Young, 2005). As such, it appears that some environments favor the active participation of juveniles in foraging tasks more than others (Hawkes et al., 1995; Tucker & Young, 2005).

Human behavior is influenced by several trade-offs, but energy is a true limiting factor. Although, it is a reasonable premise that energetic efficiency is a driving force of the behavioral decisions of hunter-gatherers (Smith, 1979; Stephens & Krebs, 1986; Ydenberg et al., 1994), it is unrealistic to expect that all human behaviors are prone to the maximization of energetic efficiency given the complexity of social, cultural, and biological constraints on humans (Bliege Bird et al., 2001; Winterhalder & Smith, 2000). If human subsistence engages high-intensity foraging tasks and high-cost activities to acquire more energy at a faster rate in less time (Kraft et al., 2021; Pyke et al., 1977), foragers would tend to maximize the energetic return instead of trying to be more efficient. Actually, several options are available to foragers intending to optimize efficiency. Several questions are still ongoing: why do juveniles make different choices? What are the acquisition rates of juveniles? And how different is their energetic cost in foraging in comparison to that of adults? Comparative data on the energetic costs of digging tubers and yucca (E_dig_) (extracted from Kraft et al., 2021: Table S6) in Hadza, Tsimane and Bwindi adult populations show an average of 4.54 kcal/hr for each kilogram of an individual’s weight (in both sexes). In the urban sample of children and adolescents studied here, the average E_dig_ is 4.01 kcal/hr per kg (in boys and girls). Thus, the range for juveniles (3.55–4.35) overlaps with the adult range (3.41–6.52), although it is slightly lower.

Therefore, children and adolescents have different levels of foraging efforts and self-provisioning tasks across different hunter-gatherer groups. In certain environments, juveniles forage quite effectively, and their foraging decisions match the predictions of the optimal foraging theory (Winterhalder & Smith, 2000). Thus, the key question is whether juveniles forage less productively than adults because they cannot do so efficiently at those ages (Gurven et al., 2006; Kaplan et al., 2000) or if they make optimal foraging decisions even though they are smaller and have less strength and ability (Kelly, 2013). Published values on children’s foraging on the Island of Mer (Bird & Bliege Bird, 2005; Bliege Bird & Bird, 2002), or the Martu of Australia (Bird & Bliege Bird, 2005), and the Mikea of Madagascar (Tucker & Young, 2005) show that juveniles (especially the youngest ones) do not try to be efficient when working (neither return rate maximizers nor time minimizers) (Bird & Bliege Bird, 2005, and references therein). However, they do forage efficiently, and this can be traced back to how they simulate foraging when playing outside the camp, as well as not being trained by adults until they are older (Crittenden, 2016; Gosso et al., 2005; Kamei, 2005). Furthermore, Meriam juveniles adapt their fishing methods to their individual strengths and their prey choice to their optimal walking speed (Bliege Bird & Bird, 2002) in order to act more efficiently. As such, this efficiency does not seem to be a question of practice or learning.

In addition, the small effect of the differences in size and strength among prepubescent individuals on the simulated digging activity observed here have implications for our knowledge of the onset of gendered foraging behaviors in cooperative societies. Research focused on the gender differences in physical activity levels and energetic expenditure during foraging in adults showed mixed results (Gurven & Hill, 2009; Marlowe, 2007). Hadza adults show a noticeable sexual division of labor and sex differences in spatial behavior and landscape. Those differences start in boys and girls at an early age (Wood et al., 2021). In contrast, in other populations, such as Amazonian Shuar, males have greater foraging energetic costs than females but both sexes have very high physical activity levels (Christopher et al., 2019; Madimenos et al., 2011). Likewise, Congo Basin BaYaka foragers differ in physical activity levels and average energetic costs by sex, but females spend more time in more intensive activities than males (Sarma et al., 2020). Our results suggest that sex-specific differences during childhood and adolescence in some foraging activities, such as digging, are not explained by differences in efficiency or energetic costs.

In summary, although foraging proficiency increases with age, it is evident that young foragers are able to obtain food on their own in an efficient manner, especially if they are part of a foraging party led by more skillful individuals. The acquisition of energetic resources depends not only on the progressive knowledge of certain skills or on body size because the energetic output of performing the task has a real influence on foraging proficiency. Although the relationship between the acquisition of complex foraging skills and the ontogenetic factors of human life history traits and cognition is still under debate (Lew-Levy et al., 2021), the effects and interactions of body size, strength, and practice on the improvement of subsistence skills remain uncertain. In our experimental approach, we claimed that digging is an energetically expensive activity, although youth of both sexes found it easy to perform in a social and recreational environment. The long nature of the juvenile period makes it possible for youth foragers to invest important physical effort into performing certain tasks, while also coping with the energetic constraints of physical growth and maturation.

## Conclusions

Despite sex-specific and age-specific differences in the net energy expenditure of digging, both girls and boys had a similar rate of skillfulness. According to the experimental evidence presented here, digging is a moderately vigorous activity for girls and boys from 8 to 14 years of age that requires significant physical effort, which is dependent on strength and body size and is closely related to age. Thus, skill-based constraints are not the only limitations for youth foragers to reach peak proficiency and productivity and to become net producers or providers. Beyond the arduousness of practicing adult skills, other factors such as body size and strength may also be important constraints for the extractive foraging efforts of juveniles. Thus, the energetic output of performing the task has a real influence on foraging efficiency.

## Supplementary Information

Below is the link to the electronic supplementary material.

Supplementary file1 (PDF 303 KB)

## Data Availability

The primary dataset used in this manuscript will be available in the online database of Mendeley Data: Mateos, A., Rodríguez, J. 2021. *Energy expenditure in children and adolescents during digging activities. Dataset from EVOBREATH 2016–2017*. Mendeley Data, v1, doi: 10.17632/rvyzmy88c5.1
