# Supplementary material for: Let’s Play at Digging: How Vigorous Is This Energetic Task for a Young Forager?
Source: Hum Nat. 2022 Jun 6;33(2):172–95. doi: 10.1007/s12110-022-09428-w (PMC9250475; doi:10.1007/s12110-022-09428-w)

Let’s Play at Digging: How Vigorous Is This Energetic Task for a Young Forager?
Ana Mateos, Guillermo Zorrilla-Revilla, and Jesús Rodríguez
*Corresponding author: Ana Mateos. ana.mateos@cenieh.es
Published in *Human Nature 33* (2022), https://doi.org/10.1007/s12110-022-09428-w

Figure S1. Graphical example of the output produced by the LabManager IntelliSupport 5.72 application on data recorded with Oxycon Mobile JAEGER® device. The example corresponds to an experimental gymkhana, including a digging trial, performed by two of the subjects participating in the study. The O_2 consumed ($V'O_2$, in blue) and the CO_2 produced ($V'CO_2$, in red) were measured, breath-by-breath, to compute the equivalent metabolic rate in kilocalories (EE, in green). The time interval corresponding to the digging trial is delimited by two vertical yellow lines (from minute 5 to minute 20). (Top) Test output from BE0243 (male, 11 years old). (Bottom) Test output from BE0315 (female, 12 years old).

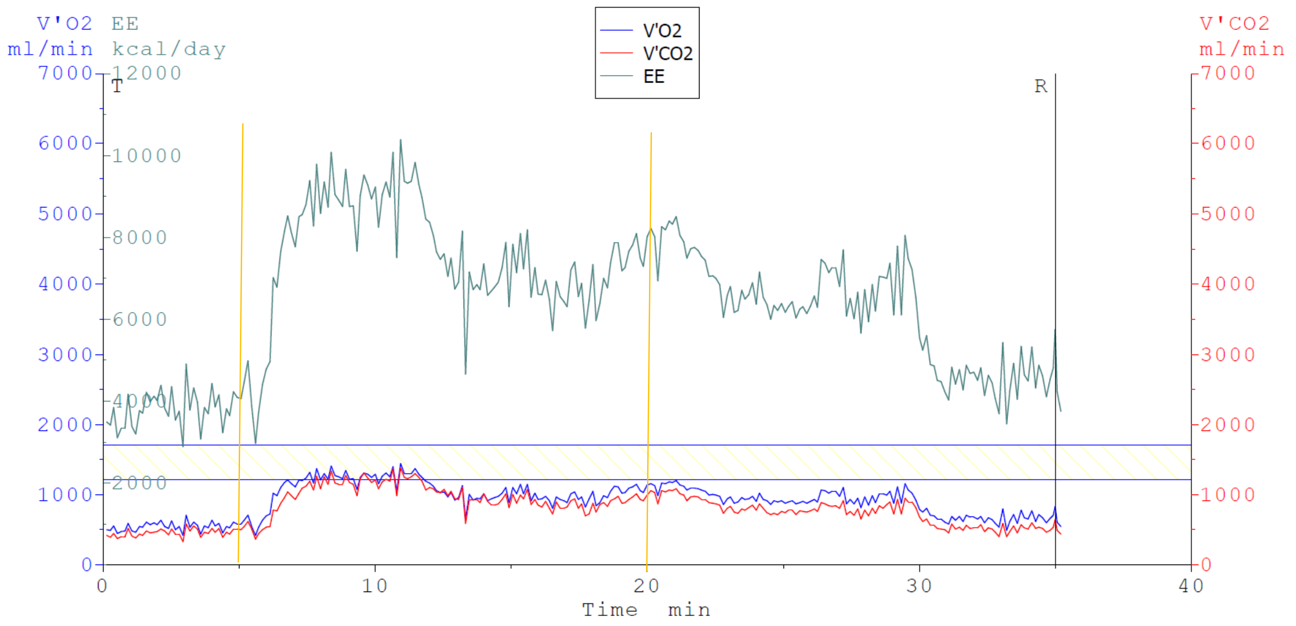

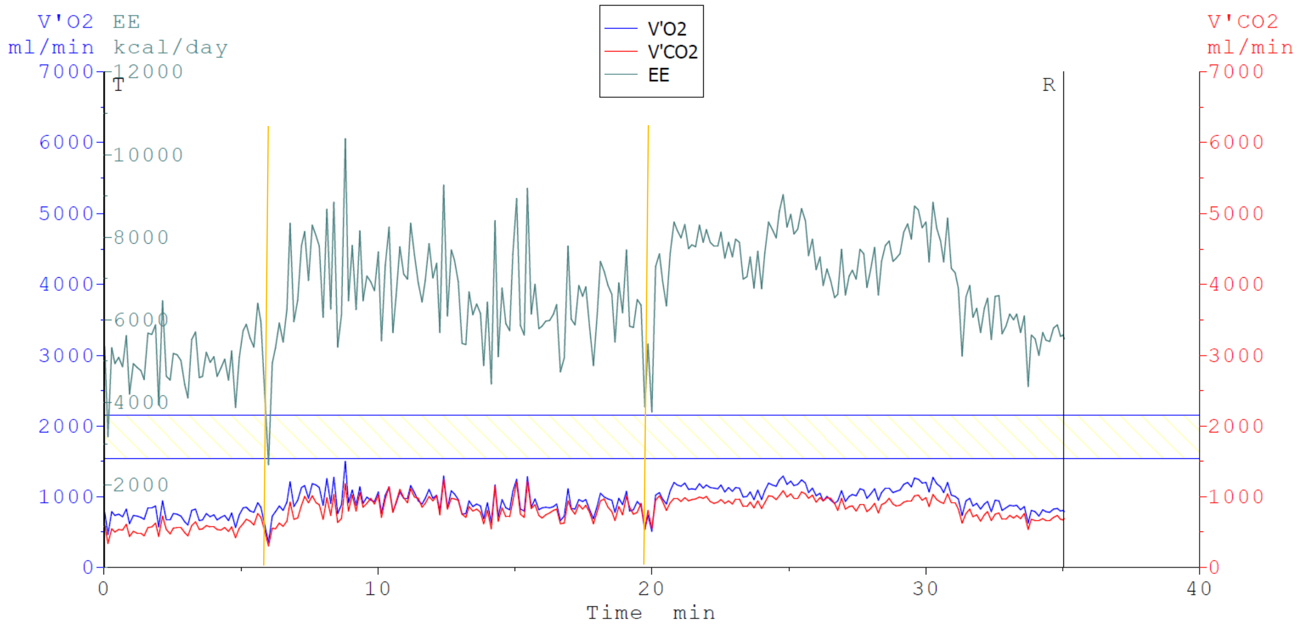

Supplement: Supplementary file 1 — Supplementary file1 (PDF 303 KB) [file 12110_2022_9428_MOESM1_ESM.pdf]
